# Experience of endometriosis pain: a qualitative study

**DOI:** 10.1097/j.pain.0000000000003763

**Published:** 2025-07-24

**Authors:** Amanda C. de C Williams, Afra Azadi, Honor McGrigor

**Affiliations:** a Research Department of Clinical, Educational & Health Psychology, University College London, London, United Kingdom

**Keywords:** Fear, Avoidance, Psychological models

## Abstract

Endometriosis is a common disorder in postpubertal women and often associated with pain in the pelvis and elsewhere. Pain and related symptoms, such as fatigue, adversely affect quality of life. Surgical and hormonal treatments have limited efficacy and can cause additional problems. There are surprisingly few studies of psychologically based interventions aimed at pain management and a better quality of life, although women with endometriosis are included within mixed chronic pain populations. As studies of pain management applied to endometriosis start to emerge, it is important to ascertain whether the assumptions of the dominant psychological model of pain, fear, and avoidance (developed mainly in musculoskeletal pain) are appropriate for the population and the problems associated with pain. We conducted a qualitative study of 16 women with endometriosis, using a very open interview technique (Grid Elaboration Method) and thematic analysis of women's accounts. We elicited 6 interrelated themes: 1 on the nature of pain; 3 on impact on everyday life, social life, and relationships, and emotions; 1 on healthcare experiences; and 1 on the burden of being female. Even when describing concerns about what was causing pain, women expressed few fears about physical integrity, nor did they avoid activities with the intention of protecting themselves from damage or increased pain. This leads us to question the applicability of the fear and avoidance model, and we encourage further exploration of the psychology of endometriosis-related pain to better design effective pain management interventions.

## Abstract 2

Supplemental Digital Content is Available in the Text.

A qualitative study of women with painful endometriosis identified few fears of pain or damage, despite extensive psychological and social impact underpinning certain avoided activities.

## 1. Introduction

Around 5% to 8% of women of reproductive age have endometriosis,^10,21,26,44^ usually with pelvic pain.^14,16^ Pain and other symptoms, such as fatigue, can be constant, unpredictable, or follow the menstrual cycle^16^; it is unrelated to disease signs such as lesions or inflammatory markers,^47^ with signs of central sensitization and co-occurrence with other persistent pain conditions, including fibromyalgia, bladder pain, and low back pain.^1,21,46^ Diagnosis is often delayed by years after onset of symptoms^21^ that are dismissed as “normal” dysmenorrhea.^7,10,35,53^ Treatment of disease or fertility problems often takes precedence over pain management, and neither surgical, hormonal, nor other treatments reliably relieve pain.^20^

As with other persistent pains, women with endometriosis experience significant decrements in quality of life, mood, relationships, and life goals.^10,12,19,27,41,45,52^ These problems may be minimized or dismissed as psychological,^51^ while the association with menstrual bleeding stigmatizes their pain.^19,45^

The psychology of persistent pain is dominated by the fear and avoidance model, a cognitive behavioral formulation in which poorly informed beliefs about the body's vulnerability to harm, and a bias toward catastrophic interpretations of interoceptive experience and external information, inform overcautious behavior, with avoidance of normal physical activities. Avoidance prevents disconfirmation of negative predictions and becomes self-maintaining, despite the loss of valued activities and roles that this entails. This model, developed over the past 20 years,^55–57^ has informed a generation of effective rehabilitative treatments.^28,31,59^ While some presentations of the model specify musculoskeletal pain,^56,57^ many do not,^9,32,54^ and it has been extended to visceral pains, where it has displaced stigmatized psychogenic formulations.^9^

It is not clear to what extent the fear and avoidance model applies to endometriosis, whether there are fears that pain implies damage, or protective avoidance of apparent triggers of pain. A meta-analysis of 335 studies testing associations between fear of pain, catastrophizing, and disability contained only 6 studies of visceral pain.^39^

Endometriosis gives grounds for serious health concerns, including compromised fertility, and mood and social adjustment are negatively affected,^36^ although neglected in qualitative research.^62^ A narrative synthesis of qualitative studies^10^ described themes of powerlessness, loneliness, and worry about infertility but not anxiety about pain. By contrast, 3 reviews^3,26,64^ and an observational study^63^ reported catastrophic thinking to be associated with more avoidance, and another observational study associated fear of disease progression and negative interpretative bias in endometriosis with greater disability.^49^

Overall, there is little theorizing in this area about the nature of distress or about the fit of existing psychological models of pain to women with painful endometriosis. This study enquired in an open-ended way into women's experience of painful endometriosis.

## 2. Methods

### 2.1. Design and setting

This study was part of a wider project investigating psychological models of pain in visceral disease (APDP ADVANTAGE: protocol for qualitative studies on https://osf.io/59d6t) and had UCL Ethical Committee approval (Ref No. 2182). Women with endometriosis were recruited through a large UK charity, Endometriosis UK (https://www.endometriosis-uk.org/), by advertising the study on their website. The method adopted was reflexive thematic analysis with inductive coding.

### 2.2. Participants

The study aimed to recruit 16 women with endometriosis: the sample size was determined by the wider APDP ADVANTAGE project of which this is part. Inclusion criteria, provided to potential participants in the information sheet, were that participants be adult women (18 years and older) with endometriosis and with chronic pain (pain every day or nearly every day for at least 3 months); that they speak English well enough for interview; that they have access to either a laptop, tablet, or smartphone with wifi connection to participate; and that they have no significant cognitive impairment. Participants of all backgrounds were invited, but the study advertisement especially welcomed participants from minority ethnic backgrounds because of their under-representation in pain research.

### 2.3. Procedure

Interested potential participants were directed to more information about the study, and a Qualtrics form on which to register their interest by completing questions on the inclusion criteria and contact details. When the researcher (H.M.) contacted them, she answered any questions, requested return of the consent form, and arranged an interview online. Instruction on the use of Microsoft Teams for the interview was provided if participants were unfamiliar with it.

Interviews were conducted online between June and September 2022 and lasted approximately an hour. Participants were requested to bring paper and pen to the interview, this being checked at the beginning. They were encouraged to make themselves comfortable and to indicate whether they preferred the camera on or off. At the end of the interview, participants were thanked for their time and given the opportunity to ask questions or provide comments on the study. They were informed that a follow-up email would contain a few questions on age, sex, ethnicity, and on pain intensity and interference (from the Brief Pain Inventory [BPI]),^11^ to be completed in their own time and returned. Participation in this study was voluntary, and no compensation was offered.

### 2.4. Interviews

Interviews were conducted using the Grid Elaboration Method (GEM),^24^ which starts with the request that the participant put in each of 4 boxes on a 2 × 2-grid 1 word, phrase, or drawing about her pain: “*Please write or draw, whatever feels most comfortable to you, any thought, feeling, or idea that comes to mind when you think about your pain, and the experiences around it, in relation to your endometriosis*.” Once complete, the participant held this grid to the camera and the interviewer took a screenshot. The interview consisted of elaboration of each item in the grid, using prompts such as “*Can you tell me (more) about x?*”, “*Would you like to add anything?*”, and, when appropriate, “*How does that make you feel?*”, until the participant had no more to add. This method, based on free association, elicits authentic personal thoughts and emotions unconstrained by interviewer questions, which are all invitations to elaborate, moving on to the next item if the participant wished.

As outlined in GEM guidelines,^24^ researchers ensured that the conversation was led by the participant, with no new content being introduced unless it was previously raised by the participant. When such concepts were elaborated on, it was in the words of the participants (eg, by saying “*You mentioned x, could you tell me a little more about that?*”). After all 4 boxes had been explored, the researcher listed the concepts back to the participant and asked whether there was anything else they wished to add. The female interviewer was trained by a research student of the originator of the technique. Interviews were audio-recorded, with participants' permission, and transcribed soon afterward, deleting identifying information. For reflexivity, the researcher also made additional notes on how she felt each interview went.

### 2.5. Researcher perspectives

It is essential for researchers to state and reflect on the position from which they approached their research. Throughout the project, researchers discussed interview findings and process, aiming for reflexive processing of data, considering how their beliefs and concerns might influence their reading and analytic decisions.

H.M. is a research assistant with experience in qualitative research and a MA in Art History and Psychology. This was her first project researching endometriosis, and she considers the biopsychosocial model to be the most convincing pain model to date. She is committed to improving women's health provision. A.A. is a research assistant who joined the project after interviews were complete and led the analysis. She was trained in GEM under the guidance of its originator during her undergraduate degree and used her training to analyze data. She has symptoms that resemble endometriosis, and, although she has not received any formal diagnosis, she was mindful of the possibility of her experiences informing results, and thus, to minimize biases, would often be in communication with the first author (A.W.) whenever there was a concern about biases interacting with data interpretation. A.W. is an academic and clinical psychologist, with more than 35 years' experience working in chronic pain, including chronic pelvic pain. While she has used the fear and avoidance model in academic and clinical work, she considers it to capture only part of the chronic pain experience, even in musculoskeletal pain; she embarked on this research with open questions about whether the model described the visceral pain experience well.

### 2.6. Data analysis

Transcribed interviews were analyzed on NVivo software using thematic analysis, the recommended method with GEM data. Thematic analysis is a systematic approach that seeks to identify, analyze, and interpret themes within the interview data.^4,23^ Transcribed participant accounts are read to familiarize researchers; coded line by line; codes inductively combined into subthemes and themes, which are reviewed and revised, with draft mapping of relationships between them; themes and subthemes named, their relationships mapped, and the final report illustrated using quotations from participants.^4^ In addition, 2 elements were added that are consistent with GEM methods.^23^ A coding frame—a tabulated representation of codes, their meanings, and example quotations—was generated, and codes were applied by a second researcher (A.W.) to 4 of the 16 transcripts and several new codes generated, while others were combined. Prevalence of themes and subthemes across participants were tabulated as salience to identify relative frequency in the sample, to better inform the provisional model of pain in endometriosis, and to constrain the influence of researcher bias in developing the model. Relationships between themes were also specified where possible.

Brief Pain Inventory data were used only to describe the population and were not analysed in relation to qualitative content.

### 2.7. Methodological rigor

As well as the coding of 4 transcripts by a second researcher, with discussion and consensus about changes to codes and subthemes, rigor of the study was maintained through regular supervision and discussion, including with the originator of GEM, and addressing the quality criteria for thematic analysis proposed by Braun and Clarke.^5^ We also provide a COREQ checklist^50^ in the supplementary files, http://links.lww.com/PAIN/C354. The provisional results were then discussed individually with several expert advisors, including the CEO of a large endometriosis charity; an expert by experience on the project advisory group; 2 gynecologists and a clinical psychologist, all of whom work clinically with endometriosis and are active researchers in the area; a physiotherapist specializing in pelvic pain and providing pain management for women with endometriosis; and one further expert by experience, a volunteer from the Endometriosis UK helpline. Notes from these discussions were used to revise and refine the thematic map and presentation of results.

## 3. Results

Sixteen women were recruited: all completed grids and interviews, and the BPI, except 1 who missed 2 items on the interference scale (these were prorated). Recruitment targets were met very rapidly and there were no refusals of eligible women, nor did any participant request withdrawal of data. The first interview was run as a pilot, but because no changes were made, it was included in the sample. Participant characteristics are summarized in Table 1. Women reported having endometriosis for a mean of 13 years (range 3-32 years), with some noting intermittent pain earlier than the current persistent pain. Only one had pain restricted to the pelvis: all other women reported pain elsewhere, particularly in the back, hip, and legs. A range of analgesics were used by 12 women, with 2 missing data; several women also noted that they used hot water bottles regularly. Thirteen of the 16 offered a comment on the interview process: All were positive, feeling heard, respected, and comfortable with describing their experiences.

**Table 1:** Characteristics of participants.

Variable	Values
Age group	Five 18-29, four 30-39, six 40-49
Ethnicity	9 White, 4 Black/Black British, 3 Asian/Asian British
Average pain: BPI 0-10	Mean 5.1, range 3-8
Worst pain: BPI 0-10	Mean 6.8, range 3-10
Interference: BPI 0-10	Mean 6.1, range 2.6-7.1
Endometriosis started	Mean 13 y ago, median 10 y, range 3-32 y
Current treatments	Nonsteroidal anti-inflammatory and/or opioid-based analgesics (6), hormones (3), recent surgery (2), SSRI antidepressants (1), anticramp preparation containing hyoscine butyl bromide (1)

Brief Pain Inventory scores for pain and pain interference were within score ranges seen in clinical populations, albeit not notably high. The highest mean interference scores were for interference of pain with mood and with enjoyment (both >7/10), and the lowest was interference of pain with walking (<5/10).

### 3.1. Thematic analysis

**Figure 1.**
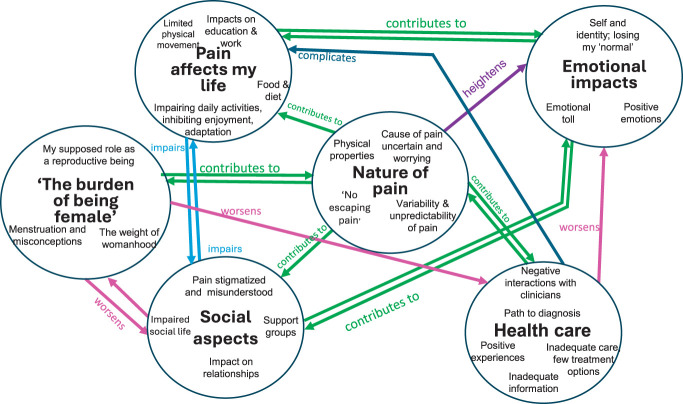
Thematic map depicting the relationship between themes and subthemes. Bold type indicates themes; subthemes are contained within the circle around each theme. Relationships between themes are shown by arrows.

Six themes were derived to describe the pain experience in endometriosis: (1) Nature of pain; (2) Emotional impacts; (3) Pain affects my life; (4) Social aspects; (5) “The burden of being female”; and (6) Health care. Table 2 summarizes identified themes, subthemes, and their saliencies, and Figure 1 presents a thematic map depicting how these themes relate to one another. Where themes have been named using the words of participants, they are represented within single inverted commas; illustrative quotations from women are given in italics and double inverted commas, with the participant number following in brackets.

**Table 2:** Saliencies of themes and subthemes.

Theme	Subtheme	Salience of themes	Salience of subthemes
		n	n
Nature of pain		16	
	Physical properties		16
	Variability and unpredictability of pain		15
	No escaping pain		13
	Cause of pain uncertain and worrying		11
Emotional impacts		14	
	Emotional toll		11
	Self and identity; losing my “normal”		13
	Positive emotions		5
Pain affects my life		16	
	Impairing daily activities, inhibiting enjoyment, adaptation		16
	Impacts on education and employment		15
	Physical movement limited		11
	Food and diet		7
Social aspects		16	
	Impact on relationships		16
	Pain stigmatized and misunderstood		16
	Impaired social life		8
	Support groups		7
The burden of being female		15	
	My supposed role as a reproductive being		11
	The weight of womanhood		9
	Menstruation and misconceptions		9
Health care		16	
	Negative interactions with clinicians		16
	Inadequate care, few treatment options		15
	Path to diagnosis		13
	Inadequate information		13
	Positive experiences		6

#### 3.1.1. Theme 1: nature of endometriosis pain

The nature of endometriosis pain heightened its Emotional impacts, not least where there was worry about the cause of pain; it contributed directly to the Pain affects my life through a range of restrictions identified in that theme, and similarly to Social aspects, particularly unpredictable pain in relation to planned social activities. The relationship with “The burden of being female” was bidirectional, in that the pain was a direct reminder of womanhood, and the meaning of pain was infused with its associations with fertility and female physiology. There was also a bidirectional relationship with Health care, in that before diagnosis, the pain was often dismissed as normal period pain, and after diagnosis, was often not adequately addressed in health care that focused instead on fertility.

##### 3.1.1.1. Physical properties

The individual nature of pain was emphasized, with diverse, often extreme, descriptions of the sensations: “*being wrapped in barbed wire*” (P10) and a “*sharp explosion*” (P16). Pain also varied across time, sometimes in relation to triggers such as diet or stress identified by women, but for most unpredictably, despite attempts to identify triggers. Flares of pain were mentioned by most women, related to ovulation, menstruation, or activity. Pain was often accompanied by fatigue and low energy, bloating, and “brain fog.”

“*So when I talk to my friends, I will have a whole conversation with them, and then, because I'm so tired, because I'm so exhausted, I don't remember the conversation very well at all. So, I think like my memory becomes really impaired as a result of being exhausted*” (P13).

##### 3.1.1.2. Variability and unpredictability of pain

All but 1 woman described pain as variable and/or unpredictable, day to day and across months and years, undermining any sense of control.

“*just when I feel like I'm getting used to how things are and suddenly things are worsening*” (P4).

##### 3.1.1.3. No escaping pain

Women felt “stuck” with pain and the emotions it elicited.

“*You're feeling this pain and there is nothing you can do about it…. It's never going to end*” (P12).

##### 3.1.1.4. Cause of pain uncertain and worrying

Given the changeability of pain described above, many participants tried to give meaning to these changes, in relation to triggers or to disease progress, or to the success or otherwise of treatment.

“*It's scary not knowing what's going on or why you're in pain or whether it's going to get worse*” (P10). “*You're just sort of like questioning all the time, what's actually happening in your body. And then questioning what the solution is to that*” (P12).

“*But I know that it's… it's coming back… Eventually it's going to get as bad as it was, probably. I don't think anything is going to be done about it anymore*” (P5).

#### 3.1.2. Theme 2 Emotional impacts

The emotional impacts of pain had a bidirectional relationship with Social aspects, with the emotional toll of pain adversely affecting social life and relationships, but with support groups generating largely positive emotions. There was also a bidirectional relationship with Pain affects my life, particularly with reduced enjoyment of previously pleasurable activities, and lack of pleasure in life depressing mood.

##### 3.1.2.1. Emotional toll

The emotional toll of endometriosis pain came partly from the day-to-day exhaustion of living with pain, but also from frustration with the lack of effective treatment. One participant even noted that she “*had breast cancer twice and felt more despair with endometriosis than cancer*” (P6). Many felt isolated, worsened by others minimizing or dismissing their pain.

*“There is no way for someone to sympathize, truly, with pain, and that's why it's so lonely”* (P13).

*“Pain is a lonely thing*” (P14).

Difficulty in understanding what was happening in their bodies to cause pain (Subtheme 3.1.1.4) exacerbated this. Women also reported feeling anxious, depressed, and hopeless; 4 mentioned having had suicidal thoughts:

“*You just can't enjoy life in the… in the same way and…everything becomes exhausting… like what's the point in this?*” (P13).

##### 3.1.2.2. Self and identity; losing my “normal”

Women described the impact of endometriosis pain on their sense of self, particularly in relation to body image, but also in relation to strain in relationships.

“*I didn't really feel like I was a proper woman… It's hard to feel sexy and attractive when you've got this ongoing pain*” (P6).

“*I've changed in a bad, bad way… I don't like the way I am now at all*” (P3).

Adjusting to endometriosis pain, particularly when it was variable and unpredictable, was demanding, causing divergence from the future women had planned.

“*I should be enjoying life… in your 20s, it's all about living life, isn't it? I just couldn't*” (P6).

##### 3.1.2.3. Positive emotions

Some participants reported positive emotions: gaining empathy, satisfaction from helping others, and from support and collective advocacy for endometriosis.

“*Know we're not invisible, that we've been listened to*” (P15).

#### 3.1.3. Theme 3: pain affects my life

This theme was closely and bidirectionally associated with Emotional impacts, as described above, with particular implications for identity, and with Social aspects, through restrictions on movement, food, energy, and other adaptations around pain and bleeding.

##### 3.1.3.1. Impairing daily activities, inhibiting enjoyment, adapting

Everyday activities, eating, sleeping, and self-care, were all affected by endometriosis pain, making activity more effortful.

“*When you're in that much pain, it's really difficult to carry out kind of normal duties… it takes like a double energy to then pick that thing off the floor. So things like housework kind of go out the window when you're in pain*” (P12).

Enjoyment was also diminished, whether of commonplace activities or of major goals.

“*I love to bake, and I can't even do that. I can't stand up long enough to do that*” (P10).

“*My pain is like a barrier to who I am, and my life*” (P13).

All women tried to plan around pain but found it hard to manage unexpected flares. Several described changes to diet, working on breathing, or toward healthier lifestyles, in an effort to control pain. A few endorsed the identity of disability, arguing that this could improve recognition of problems associated with endometriosis.

“*I know at times in my life, it has been like very debilitating and it would be helpful for me to like be able to give people a piece of solid information that they understand and I think that people understand the word 'disabled'*” (P16).

##### 3.1.3.2. Impacts on education and employment

School and university attendance and capacity to study were affected by pain, sometimes to the point of withdrawal, as was employment: travelling between worksites, having to stand (such as to teach or present work), illness absence resulting in threats to employment, and punitive behavior from managers or workmates. Some participants had resorted to working part-time, with the financial penalties associated, or limiting their careers in some way.

“*There's part of me thinking that I don't know if I should be even in construction, but I'm here for the moment*” (P7).

However, women also minimized the benefits of employment, with some expressing gratitude for understanding employers, and others finding a solution by becoming self-employed.

“*If I've got a really bad period for two or three days, there's no need for me to be sat in an office, in pain. I can work from home*” (P12).

##### 3.1.3.3. Physical movement limited

This varied from extremes such as being bed-bound to being unable to take vigorous exercise.

“*In 2021, I was actually bedridden for three months, before I got the implant, because I was just in so much pain. I couldn't even like go on five-minute walks*” (P10).

##### 3.1.3.4. Food and diet

This was a widely described trigger, but restricting food or food types (most often gluten and caffeine, or adopting a FODMAP diet recommended for irritable bowel syndrome) could be isolating,

“*So I sort of have to decide, you know… is this food worth the pain I'm going to have?*” (P15).

#### 3.1.4. Theme 4: Social aspects

Close, and particularly intimate, relationships in this theme were affected by participants' view of their womanhood (“The burden of being female”), and in turn affected those views. There was also a bidirectional relationship with Pain affects my life, particularly around stigma encountered both in social and work or study settings. It was also, as described above, related to Social aspects.

##### 3.1.4.1. Impact on relationships

Relationships with spouses, partners and potential partners, family members, and friends were all described as adversely affected by endometriosis pain, although they also provided significant support. Most women had partners, family, or friends who had tried to understand the condition, sought information about it, encouraged them to seek treatment, and tried to accommodate to their limitations. In particular, family members who themselves had endometriosis tended to be more understanding and to help guide the participant in treatment decisions. However, women described having *“no patience”* with their children or feeling too exhausted to be as involved as they wished in their families.

*“[I] don't have much to offer because… if I'm not in bed, I'm on the sofa, horizontal… I'm off my head on the strongest medication…”* (P6).

Friendships could be strained:

“*They* [friends]*… don't have much experience in it, it's difficult for them to put themselves in my shoes*” (P1).

Uncertainty about fertility and pain on sexual penetration, coupled with negative feelings about their bodies, made for difficulties in loving relationships.

“*I'm in a healthy relationship, everything should be good… it shouldn't be painful*” (P14).

Some women tried to hide their pain from their partners during sexual activity, but others felt that their partners were unable to understand, creating an emotional barrier. Similar problems arose in dating or starting a new relationship:

“…*to have to explain all of this stuff. It's just…* [gestures brain exploding] *You know? But then I want the relationship. You know, I feel lonely and it's kind of like I miss all that. So….* [crying] *I hate what it's done*” (P6).

##### 3.1.4.2. Pain stigmatized and misunderstood

Stigma was emphasised by participants as an unnecessary extra problem that they faced in managing endometriosis and pain, having found their pain disbelieved, minimized, and dismissed in everyday life and in healthcare settings. The impact was particularly noted by women from ethnic minority backgrounds.

“[You feel] *worthless… knowing there is going to be no change in your situation because he…. Or she hasn't believed you*” (P15).

*“I've been to the doctors, they all said it's normal, the media said it's normal. You'd speak to your friends. They're all like, yeah, a little bit of pain is normal. Pain is normal. I spoke to my mum as well. She was like, yeah, pain is normal. I spoke to other elders, like other aunts and things, they're all like, yeah, it's normal. And I was like, but what I'm feeling doesn't sound normal”* (P2).

Stigma was exemplified by the difficulty talking openly about endometriosis pain, which in turn discouraged participants from expressing their pain in social interactions, and even when talking to healthcare staff.

*“*[People] *don't want to hear it… it's too taboo to talk about, like, women's stuff… I remember being in a room, guys talking about their vasectomies, and that was fine”* (P7).

##### 3.1.4.3. Impaired social life

Attending social events or meeting friends was difficult and liable to be cancelled, making participants feel left out. Even when in social settings, women could feel different.

“*My friends are always like going out and doing things, and I can't, a lot of the time*” (P10).

*“*[I'm] *aware of* [my] *body while everyone else is… fully absorbed”* (P14).

##### 3.1.4.4. Support groups

Both websites and support groups provided information, shared experience, and gave a sense of community.

“*Even though it sucks to see that so many people are going through it, it's just nice to have, you know, a very safe community*” (P11).

#### 3.1.5. Theme 5: “the burden of being female”

As described under the theme of Nature of pain, the meaning and implications of endometriosis pain informed the experience of pain. There was a strong relationship with Health care, where encountering misogynistic and dismissive attitudes strengthened women's sense of unfairness based on sex and contributed to negative interactions with clinicians. Furthermore, the stigma and lack of understanding that characterized some Social aspects contributed further to this sense of unfairness.

##### 3.1.5.1. My supposed role as a reproductive being

Uncertain fertility affected women in multiple ways: in their sense of being complete women; in relationships that had assumed the possibility of a family; and in treatment options and decisions.

“*What they all ask you straight away is what you want to do about your fertility? And I think treatment then stems from what you say about that*” (P4).

*“I don't fit the traditional mold of what it is to be a woman because of my condition”* (P13).

##### 3.1.5.2. The weight of womanhood

Misogyny that contributed to difficulties establishing their pain as a medical problem was described particularly by women from ethnic minorities.

“*You're a woman, that* [the pain] *is supposed to happen*” (P5).

*“As a Black woman, we do have different experiences to other women, and sadly, some of them can be of a more negative persuasion… there's this whole thing that, we are seen to be, you know, we can handle pain better… But the fact is it means that sometimes we could be more overlooked”* (P6).

##### 3.1.5.3. Menstruation and misconceptions

The universality in women of period pain at some level of intensity meant that women's endometriosis-related pain was often dismissed, while recognizing how hard it is to compare pain between people.

“*Obviously comparing to my friends… I never understood how mine would go on for so long. I know it's very different for every woman*” (P11).

#### 3.1.6. Theme 6: Health care

Adverse experiences in health care formed an important part of some women's narratives and contributed to the emotional toll of living with endometriosis and pain (Emotional impacts). The bidirectional relationship with the Nature of pain also consisted of comments on the ways in which variability and unpredictability of pain seemed to undermine clinicians' confidence in diagnosing and treating endometriosis. Experience of health care also contributes to Pain affects my life, adversely in several participants through side effects of medication, and in others because of the inadequacy of treatments to resolve pain and other problems that affected home and work life.

##### 3.1.6.1. Negative interactions with clinicians

Unsupportive and dismissive clinicians had been encountered by most women, who felt disbelieved or written off as “*being dramatic*” (P8). They were critical of the poor level of understanding and even of knowledge about endometriosis of some clinicians, particularly male doctors.

##### 3.1.6.2. Inadequate care, few treatment options

Problems arose for women at all stages from seeking a referral to specialist care, obtaining a diagnosis, getting investigations, and being offered treatment. This was in part based on their symptoms being minimized, as described in 3.1.6.1, but also (in the National Health Service), the long wait for appointments and procedures, and likelihood of cancellation. In response, several had sought private health care at considerable expense.

*“*[I had to] *fight just to get seen and spoken to, to get appointments, to get medicated, and to get an operation”* (P7).

Most women had tried contraceptive tablets and/or analgesics but reported various adverse effects that almost outweighed the benefits, although a few had gained worthwhile control over pain. Treatment seemed to consist of few options, none of them particularly satisfactory.

“*They're not a solution, it's just putting a plaster over the issue”* (P8).

Several had experienced complications of surgery, some requiring further surgery, and were critical of the lack of follow-up. They were also aware that even “*full hysterectomies…* [may bring about] *no change in their pain*” (P4). The lack of effective and acceptable treatments meant that many turned to alternatives such as “*ayurvedic doctors*” (P11), cannabinoids, acupuncture, yoga, and osteopathy.

##### 3.1.6.3. Path to diagnosis

Obtaining a diagnosis was for many women a long and arduous process, taking years, but without a diagnosis their pain and other symptoms were not taken seriously. Diagnosis itself could be very validating, but led to speculation about whether earlier diagnosis might not have made for a better treatment outcome.

“*It was like I haven't made this all up in my head; it's not because I'm bad at looking after myself. It was just like years of pain and discomfort had all been explained in some way. It's almost like rewriting your whole life… Like everything starts falling into place and making sense*” (P14).

For others, the diagnosis was unwelcome news, particularly once women became better informed about endometriosis.

*“*[I felt] *more depressed… because now I know there is no cure”* (*P4*).

##### 3.1.6.4. Inadequate information

Many women described receiving what they considered to be inadequate or incorrect information about endometriosis, with conflicting advice about managing pain, and little discussion of the advantages and disadvantages of each treatment option, meaning that decisions on treatment were poorly informed. Women tended to do their own research to feel more able to seek and make decisions about treatment and had found endometriosis organisations particularly helpful. Several commented on the lack of priority for research, given the prevalence of endometriosis.

*“I don't know how that's going to be able to be changed, but if more of us can speak about it, maybe something can be done about it sooner rather than later”* (P6).

##### 3.1.6.5. Positive experiences in health care

When women felt heard and validated, or received effective treatment, it markedly improved their capacity to manage pain, and they described with gratitude staff's efforts to treat them with kindness.

## 4. Discussion

The themes and subthemes identified were not unexpected, and the clinicians and women whom we consulted, who had endometriosis, and/or worked with women with endometriosis, largely endorsed them, with a few concerns noted in Limitations below. All but The burden of being female could arise from any qualitative study of the experience of chronic pain from endometriosis^10,12,45,52^ or other chronic pain.^6,17,52^ Some subthemes, such as effects on body image and stigma, have been found in studies of women with endometriosis that do not focus on pain.^42^ Eleven of our 16 participants did express fears and concerns about what might be causing their everyday pain, and/or flares, but without catastrophic speculations on what the pain might mean; there were no accounts of damage envisaged as the cause of pain, nor of endometriosis affecting the integrity or function of any organs in the pelvic cavity. Nor did these fears generate avoidance of particular movements, activities, or physical demands, as they commonly do in musculoskeletal pain^2,30^; avoidance was much more associated with social situations, where pain (and often copious bleeding) made it hard for women to join in. However, avoidance had no obvious protective intention in relation to the pain, as it often does in musculoskeletal pain.^2,57,58^ Ratings of pain interference (from the BPI) similarly indicated greater emotional than physical impact of pain. These findings are somewhat surprising in the light of accounts of catastrophic imagery in pain from endometriosis,^18^ fear of progression of the disease,^49^ and the association of interpretation biases in relation to pain with interference of pain with daily life.^37^

In this sense, our concerns that the fear and avoidance model should not be uncritically extended to all chronic pains, specifically to endometriosis pain, were supported by our data. There are several possible reasons for this. The most important is that we recruited from a patient charity to which women usually turn after a diagnosis of endometriosis; they might already be reasonably well-informed about the implications of their pain and have accepted the diagnosis and adjusted to it, to a greater or lesser extent. However, our thematic synthesis of 33 qualitative studies of women with pain from endometriosis^60^ reviewed studies that recruited equally from community and from clinical settings, and also found no strong evidence of fears that pain implied damage or harm, resulting in avoidance to mitigate such possibilities. A study of women with endometriosis who reported benefit from pain neuroscience education found themes of a sense of validation and a greater understanding of pain mechanisms, including psychological contributors^29^; again there was no strong theme concerning fear of pain or damage. A second possible explanation is that our methods, although very open-ended, and despite our efforts at reflexivity, affected analysis both of these interviews and the related review.^60^ In particular, our interest in questioning the applicability of the fear and avoidance model to endometriosis pain could have fundamentally affected both data collection and analysis. Both these are discussed under Limitations, below.

A third possibility is that receiving a recognized diagnosis, which many people with musculoskeletal pain seek but never obtain, resolves some uncertainties about the pain and its implications, and enables concerns to be moderated by the healthcare team. Clinicians whom we consulted about our results, and 1 expert by experience, more familiar with encountering women at the point of diagnosis and afterward, commented on seeing fewer accounts than they expected in our thematic map of anger and unfairness, of sexual problems, and of fears of compromised fertility, but they were not particularly surprised by our findings concerning few reports of fear of pain. Women with endometriosis, also consulted about our results, emphasized that our participants were all postdiagnosis, describing the achievement of diagnosis as a relief, although while it initially brings hopes of effective treatment, those hopes fade and give way to continued management of the problems experienced before diagnosis, dilemmas over the pros and cons of possible treatments, and adjustment of lifestyle. They also believed that the normality of menstrual pain and bleeding among girls and women provided fewer grounds for fears about sinister internal causes of pain than do novel musculoskeletal pains.

### 4.1. Strengths and limitations

We consider the GEM to have substantial strengths as a method for eliciting participants' viewpoints in as nondirective a way as possible. However, it may mean that more personal topics, such as sexual problems, were raised less than had we asked direct questions; women did describe sexual difficulties, such as finding positions that minimized pain, but we incorporated these in the subtheme of impact on relationships. Fear and avoidance may not have been accessible for participants to report; avoidance is very hard to capture by any means of assessment. It is also possible that, although the interviewer only asked for elaborations on each of the words, phrases, or pictures in the grid, subtle and social influences were exerted on what participants chose to elaborate. Similarly, although we tried to be sensitive to expressions of worry or fear during analysis, we may have failed to identify relevant data. Sampling from an endometriosis charity, as discussed above, necessarily underrepresented women who had not been diagnosed, but their inclusion would have cast doubt on the homogeneity of the population in relation to having endometriosis pain, rather than undiagnosed pelvic pain, and, as is evident from data, several were in active treatment pathways, so were not unlike patients sampled from clinical sources.

### 4.2. Implications

The implications of this work mainly concern health care, and many are shared with major reviews^48,61^ and inquiries,^33^ which find generally poor standards of care, with prolonged waits for diagnosis after failures to refer to specialist care, a preoccupation with fertility at the expense of pain and quality of life, and marked inequalities of access and care.^34,48^ Both the latter attribute care shortcomings to sexism, racism, and unconscious bias, and call for a change in the culture around women and periods, in particular during school years where better education could enable girls to have more confidence in deciding what symptoms are abnormal. The endometriosis charity which helped us in this research clearly provided valued support for women, and to an extent, this filled some gaps in health care.

The inquiries and reviews also assert the need for multidisciplinary care, including pain specialists and psychological therapists.^33^ A systematic review of psychological interventions for endometriosis found 7 randomized controlled trials of somewhat eclectic treatments often aimed at symptoms other than pain; it reported benefits for some physical symptoms and for mental health, but not for physical or social function.^13^ Another systematic review of biopsychosocial approaches to pelvic pain, although not for endometriosis, reported some benefits for pelvic pain and emotional outcomes.^25^ However, given the nature of the pain,^8^ there is no reason not to test the principal components of psychologically based treatments in more rigorous trials. In trials of physical treatments, mainly surgery or hormones, changes in pain, function, and distress are often poorly evaluated,^8,38^ leading to calls for an agreed outcome set for endometriosis interventions.^40^ As in other areas of evaluation of treatment for chronic pain, passive collection of data by digital technologies such as smartwatches is underused.^15^ Social outcomes are inadequately addressed in research, particularly stigma,^45^ although they are important in withdrawal from social activity and problems at work or school; a few studies address the effects on partners of women with endometriosis^22,43^ and show the complex effects of endometriosis on sexual intimacy, and on plans for a family.

There are various research implications other than replication. Following through the ideas raised here could be performed in studies recruiting from clinical populations; collecting more detail of current and past medical care in relation to adjustment to endometriosis pain; and direct assessment of catastrophic thinking and fear of pain, without personal contact as in interviews.

## 5. Conclusions

We did not find support for the assumption that the dominant psychological model in chronic pain applies in endometriosis. This model, fear and avoidance,^9,57^ describes overestimation of and catastrophic interpretation of the threat inherent in pain for physical integrity, leading to avoidance of a wide range of activities, the avoidance being intended to protect from further pain and injury, but in reality leading to both increasing disability (through deconditioning) and depression (through losses inherent in avoidance). However, women found pain disruptive of a wide range of activities through its general effects, with avoidance of particular situations, not least social ones, related to perceived stigma, practical concerns about concealing pain and bleeding, and to related symptoms such as fatigue and bloating. Many of our findings about the impact of endometriosis and pain on women's lives are shared with other studies,^60^ but the psychological formulation of these problems is novel and invites further exploration.

## Conflict of interest statement

The authors have no conflicts of interest to declare.

## Supplementary Material

## Appendix A. Supplemental digital content

Supplemental digital content associated with this article can be found online at http://links.lww.com/PAIN/C354.

## References

[1] Aredo JV Heyrana KJ Karp BI Shah JP Stratton P. Relating chronic pelvic pain and endometriosis to signs of sensitization and myofascial pain and dysfunction. Semin Reprod Med 2017;35:88–97. 28049214 10.1055/s-0036-1597123 PMC5585080

[2] Asmundson GJG Vlaeyen JWS Crombez G. Understanding and treating fear of pain. Oxford: Oxf Univ Press, 2004.

[3] Brasil DL Montagna E Trevisan CM La Rosa VL Laganà AS Barbosa CP Bianco B Zaia V. Psychological stress levels in women with endometriosis: systematic review and meta-analysis of observational studies. Minerva Med 2020;111:90–102. 31755674 10.23736/S0026-4806.19.06350-X

[4] Braun V Clarke V. Using thematic analysis in psychology. Qual Res Psychol 2006;3:77–101.

[5] Braun V Clarke V. One size fits all? What counts as quality practice in (reflexive) thematic analysis? Qual Res Psychol 2021;18:328–52.

[6] Bunzli S Watkins R Smith A Schütze R O'Sullivan P. Lives on hold: a qualitative synthesis exploring the experience of chronic low back pain. Clin J Pain 2013;29:907–16. 23370072 10.1097/AJP.0b013e31827a6dd8

[7] Clauw DJ Essex MN Pitman V Jones KD. Reframing chronic pain as a disease, not a symptom: rationale and implications for pain management. Postgrad Med 2019;131:185–98. 30700198 10.1080/00325481.2019.1574403

[8] Coxon L Demetriou L Vincent K. Current developments in endometriosis-associated pain. Cell Rep Med 2024;5:101769. 39413731 10.1016/j.xcrm.2024.101769 PMC11513828

[9] Crombez G Eccleston C Van Damme S Vlaeyen JWS Karoly P. Fear-avoidance model of chronic pain: the next generation. Clin J Pain 2012;28:475–83. 22673479 10.1097/AJP.0b013e3182385392

[10] Culley L Law C Hudson N Denny E Mitchell H Baumgarten M Raine-Fenning N. The social and psychological impact of endometriosis on women's lives: a critical narrative review. Hum Reprod Update 2013;19:625–39. 23884896 10.1093/humupd/dmt027

[11] Daut RL Cleeland CS Flanery RC. Development of the Wisconsin Brief Pain Questionnaire to assess pain in cancer and other diseases. PAIN 1983;17:197–210. 6646795 10.1016/0304-3959(83)90143-4

[12] Della Corte L Di Filippo C Gabrielli O Reppuccia S La Rosa VL Ragusa R Fichera M Commodari E Bifulco G Giampaolino P. The burden of endometriosis on women's lifespan: a narrative overview on quality of life and psychosocial wellbeing. Int J Environ Res Public Health 2020;17:4683. 32610665 10.3390/ijerph17134683 PMC7370081

[13] Del Pino-Sedeño T Cabrera-Maroto M Abrante-Luis A González-Hernández Y Ortíz Herrera MC. Effectiveness of psychological interventions in endometriosis: a systematic review with meta-analysis. Front Psychol 2024;15:1457842. 39529727 10.3389/fpsyg.2024.1457842 PMC11551779

[14] Denny E Mann CH. A clinical overview of endometriosis: a misunderstood disease. Br J Nurs 2007;16:1112–6. 10.12968/bjon.2007.16.18.27503 18073680

[15] Edgley K Horne AW Saunders PTK Tsanas A. Symptom tracking in endometriosis using digital technologies: knowns, unknowns, and future prospects. Cell Rep Med 2023;4:101192. 37729869 10.1016/j.xcrm.2023.101192 PMC10518625

[16] Fauconnier A Staraci S Huchon C Roman H Panel P Descamps P. Comparison of patient- and physician- based descriptions of symptoms of endometriosis: a qualitative study. Hum Reprod 2013;28:2686–94. 23900205 10.1093/humrep/det310

[17] Froud R Patterson S Eldridge S Seale C Pincus T Rajendran D Fossum C Underwood M. A systematic review and meta-synthesis of the impact of low back pain on people's lives. BMC Musculoskelet Disord 2014;15:50. 24559519 10.1186/1471-2474-15-50 PMC3932512

[18] Graham CJ Brown SL Vincent K Horne AW. International survey confirms that women with endometriosis-associated pain experience a high prevalence of pain imagery and coping imagery. Eur J Obstet Gynecol Reprod Biol 2020;244:203–4. 31748149 10.1016/j.ejogrb.2019.10.030

[19] Gstoettner M Wenzl R Radler I Jaeger M. “I think to myself ‘Why now?’”—a qualitative study about endometriosis and pain in Austria. BMC Women Health 2023;23:409. [Correction 28 Aug 2023: Wenzl R, Radler I]. 10.1186/s12905-023-02576-w PMC10403941 37542309

[20] Hansen S Sverrisdóttir UÁ Rudnicki M. Impact of exercise on pain perception in women with endometriosis: a systematic review. Acta Obstet Gynecol Scand 2021;100:1595–601. 33999412 10.1111/aogs.14169

[21] Horne AW Missmer SA. Pathophysiology, diagnosis, and management of endometriosis. BMJ 2022;379:e070750. 36375827 10.1136/bmj-2022-070750

[22] Hudson N Culley L Law C Mitchell H Denny E Raine-Fenning N. “We needed to change the mission statement of the marriage”: biographical disruptions, appraisals and revisions among couples living with endometriosis. Sociol Health Illn 2016;38:721–35. 26679773 10.1111/1467-9566.12392

[23] Joffe H. Thematic analysis. In: Harper H Thompson AR, editors. Qualitative research methods in mental health and psychotherapy. Wiley Online Books, 2012. p. 209–23. doi: 10.1002/9781119973249.ch15

[24] Joffe H Elsey JW. Free association in psychology and the grid elaboration method. Rev Gen Psychol 2014;18:173–85.

[25] Johnson S Bradshaw A Bresnahan R Evans E Herron K Hapangama DK. Biopsychosocial approaches for the management of female chronic pelvic pain: a systematic review. BJOG 2025;132:266–77. online version. 39462817 10.1111/1471-0528.17987 PMC11704080

[26] Kalfas M Chisari C Windgassen S. Psychosocial factors associated with pain and health-related quality of life in endometriosis: a systematic review. Eur J Pain 2022;26:1827–48. 35802060 10.1002/ejp.2006 PMC9543695

[27] Leuenberger J Kohl Schwartz AS Geraedts K Haeberlin F Eberhard M von Orellie S Imesch P Leeners B. Living with endometriosis: comorbid pain disorders, characteristics of pain, and relevance for daily life. Eur J Pain 2022;26:1021–38. 10.1002/ejp.1926 PMC9306988 35184363

[28] Main CJ Sullivan MJ Watson PJ. Pain management: practical applications of the biopsychosocial perspective in clinical and occupational settings. Edinburgh, UK: Churchill Livingston, 2007.

[29] Mardon AK Chalmers J Heathcote LC Curtis L-A Freedman L Malani R Parker R Neumann PB Moseley GL Leake HB. “I wish I knew then what I know now”—pain science education concepts important for female persistent pelvic pain: a reflexive thematic analysis. PAIN 2024;165:1990–2001. 38452219 10.1097/j.pain.0000000000003205

[30] Martinez-Calderon J Flores-Cortes M Morales-Asencio JM Luque-Suarez A. Pain-related fear, pain intensity and function in individuals with chronic musculoskeletal pain: a systematic review and meta-analysis. J Pain 2019;20:1394–415. 31063874 10.1016/j.jpain.2019.04.009

[31] McCracken LM Samuel VM. The role of avoidance, pacing, and other activity patterns in chronic pain. PAIN 2007;130:119–25. 17240065 10.1016/j.pain.2006.11.016

[32] Meulders A. From fear of movement-related pain and avoidance to chronic pain disability: a state-of-the-art review. Curr Opin Behav Sci 2019;26:130–6.

[33] NCEPOD National Confidential Enquiry into Patient Outcome & Death NCEPOD. A long and painful road. London: National Confidential Enquiry into Patient Outcome and Death; https://www.ncepod.org.uk/endometriosis.html (2024, Accessed May 09, 2025).

[34] Perro D Seglah H Abrahams V Weckesser A Griffith VAS. Black women's menstrual and reproductive health: a critical call for action in the UK. BMJ 2022;379:o3052. 36549688 10.1136/bmj.o3052

[35] Pettersson A Berterö CM. How women with endometriosis experience health care encounters. Womens Health Rep 2020;1:529–42. 10.1089/whr.2020.0099 PMC7785068 33786519

[36] Peveler R Edwards J Daddow J Thomas E. Psychosocial factors and chronic pelvic pain: a comparison of women with endometriosis and with unexplained pain. J Psychosom Res 1996;40:305–15. 8861127 10.1016/0022-3999(95)00521-8

[37] Pickup B Sharpe L Todd J. Interpretation bias in endometriosis-related pain. PAIN 2023;164:2352–7. 37326698 10.1097/j.pain.0000000000002946

[38] Rempert AN Rempert TH Liu A Hernández A Blanck J Segars J Singh B. A systematic review of the psychosocial impact of endometriosis before and after treatment. Reprod Sci 2024;31:1828–60. 38512699 10.1007/s43032-024-01515-w PMC11216884

[39] Rogers AH Farris SG. A meta-analysis of the associations of elements of the fear-avoidance model of chronic pain with negative affect, depression, anxiety, pain-related disability and pain intensity. Eur J Pain 2022;26:1611–35. 35727200 10.1002/ejp.1994 PMC9541898

[40] Rosenberger DC Mennicken E Schmieg I Medkour T Pechard M Sachau J Fuchtmann F Birch J Schnabel K Vincent K Baron R Bouhassira D Pogatzki-Zahn EM. A systematic literature review on patient-reported outcome domains and measures in nonsurgical efficacy trials related to chronic pain associated with endometriosis: an urgent call to action. PAIN 2024;165:2419–44. 38968394 10.1097/j.pain.0000000000003290 PMC11474936

[41] Rush G Misajon R. Examining subjective wellbeing and health-related quality of life in women with endometriosis. Health Care Women Int 2018;39:303–21. 29095116 10.1080/07399332.2017.1397671

[42] Sayer-Jones K Sherman KA. “My body…tends to betray me sometimes”: a Qualitative Analysis of Affective and Perceptual Body Image in Individuals Living with Endometriosis. Int J Behav Med 2023;30:543–54. 36074337 10.1007/s12529-022-10118-1 PMC9454389

[43] Schick M Germeyer A Böttcher B Hecht S Geiser M Rösner S Eckstein M Vomstein K Toth B Strowitzki T Wischmann T Ditzen B. Partners matter: the psychosocial well-being of couples when dealing with endometriosis. Health Qual Life Outcomes 2022;20:86. 35643578 10.1186/s12955-022-01991-1 PMC9148469

[44] Shafrir AL Farland LV Shah DK Harris HR Kvaskoff M Zondervan K Missmer SA. Risk for and consequences of endometriosis: a critical epidemiologic review. Best Pract Res Clin Obstet Gynaecol 2018;51:1–15. 30017581 10.1016/j.bpobgyn.2018.06.001

[45] Sims OT Gupta J Missmer SA Aninye IO. Stigma and endometriosis: a brief overview and recommendations to improve psychosocial well-being and diagnostic delay. Int J Environ Res Public Health 2021;18:8210. 34360501 10.3390/ijerph18158210 PMC8346066

[46] Sinaii N Cleary SD Ballweg ML Nieman LK Stratton P. High rates of autoimmune and endocrine disorders, fibromyalgia, chronic fatigue syndrome and atopic diseases among women with endometriosis: a survey analysis. Hum Reprod 2002;17:2715–24. 12351553 10.1093/humrep/17.10.2715

[47] Stratton P Berkley KJ. Chronic pelvic pain and endometriosis: translational evidence of the relationship and implications. Hum Reprod Update 2011;17:327–46. 21106492 10.1093/humupd/dmq050 PMC3072022

[48] The Lancet. Endometriosis: addressing the roots of slow progress. Lancet 2024;404:1279. 39368831 10.1016/S0140-6736(24)02179-2

[49] Todd J Pickup B Coutts-Bain D. Fear of progression, imagery, interpretation bias, and their relationship with endometriosis pain. PAIN 2023;164:2839–44. 37530656 10.1097/j.pain.0000000000003003

[50] Tong A Sainsbury P Craig J. Consolidated criteria for reporting qualitative research (COREQ): a 32-item checklist for interviews and focus groups. Int J Qual Health Care 2007;19:349–57. 17872937 10.1093/intqhc/mzm042

[51] Toye F Seers K Barker K. A meta-ethnography of patients' experiences of chronic pelvic pain: struggling to construct chronic pelvic pain as “real”. J Adv Nurs 2014;70:2713–27. 25081990 10.1111/jan.12485

[52] Toye F Seers K Hannink E Barker K. A mega-ethnography of eleven qualitative evidence syntheses exploring the experience of living with chronic non-malignant pain. BMC Med Res Methodol 2017;17:116. 28764666 10.1186/s12874-017-0392-7 PMC5540410

[53] Van der Zanden M de Kok L Nelen WLDM Braat DDM Nap AW. Strengths and weaknesses in the diagnostic process of endometriosis from the patients' perspective: a focus group study. Diagnosis 2021;8:333–9. 34318653 10.1515/dx-2021-0043

[54] Vlaeyen JWS Crombez G. Fear and pain. PAIN Clin Updates 2007;XV:1–4.

[55] Vlaeyen JWS Crombez G Linton SJ. The fear-avoidance model of pain. PAIN 2016;157:1588–9. 27428892 10.1097/j.pain.0000000000000574

[56] Vlaeyen JWS Kole-Snijders AMJ Rotteveel A Ruesink R Heuts PHTG. The role of fear of movement/(re)injury in pain disability. J Occup Rehabil 1995;5:235–52. 24234727 10.1007/BF02109988

[57] Vlaeyen JWS Linton SJ. Fear-avoidance and its consequences in chronic musculoskeletal pain: a state of the art. PAIN 2000;85:317–32. 10781906 10.1016/S0304-3959(99)00242-0

[58] Vlaeyen JWS Linton SJ. Fear-avoidance model of chronic musculoskeletal pain: 12 years on. PAIN 2012;153:1144–7. 22321917 10.1016/j.pain.2011.12.009

[59] Williams ACdC Fisher E Hearn L Eccleston C. Evidence-based psychological interventions for adults with chronic pain: precision, control, quality, and equipoise. PAIN 2021;162:2149–53. 33769362 10.1097/j.pain.0000000000002273

[60] Williams ACdC McGrigor H. A thematic synthesis of qualitative studies and surveys of the psychological experience of painful endometriosis. BMC Womens Health 2024;24:50. 38238741 10.1186/s12905-023-02874-3 PMC10795225

[61] Women's health strategy for England—GOV.UK. Available at: www.gov.uk. Accessed May 09, 2025.

[62] Young K Fisher J Kirkman M. Women's experiences of endometriosis: a systematic review and synthesis of qualitative research. J Fam Plann Reprod Health Care 2015;41:225–34. 25183531 10.1136/jfprhc-2013-100853

[63] Zarbo C Brugnera A Dessì V Barbetta P Candeloro I Secomandi R Betto E Malandrino C Bellia A Trezzi G Rabboni M Compare A Frigerio L. Cognitive and personality factors implicated in pain experience in women with endometriosis: a mixed-method study. Clin J Pain 2019;35:948–57. 31433322 10.1097/AJP.0000000000000757

[64] Zarbo C Brugnera A Frigerio L Malandrino C Rabboni M Bondi E Compare A. Behavioral, cognitive, and emotional coping strategies of women with endometriosis: a critical narrative review. Arch Womens Ment Health 2018;21:1–13. 28932912 10.1007/s00737-017-0779-9

