# Supplementary material for: Experience of endometriosis pain: a qualitative study
Source: Pain. 2025 Jul 24;167(2):273–83. doi: 10.1097/j.pain.0000000000003763 (PMC12794361; doi:10.1097/j.pain.0000000000003763)
Supplement: Supplementary file 1 [file jop-167-273-s001.docx]

**Supplementary material**

**COREQ checklist**

| ***Item*** | ***Guide questions/description*** | ***Reported on page #*** |
| --- | --- | --- |
| ***Domain 1 Research team & reflexivity*** | | |
| *Personal characteristics* | | |
| 1. Interviewer/ facilitator | Which author/s conducted the interview or focus group? | HM, third author: pp 3-5 |
| 2. Credentials | What were the researcher’s credentials? E.g. PhD, MD | P 5 |
| 3. Occupation | What was their occupation at the time of the study? | P 5 |
| 4. Gender | Was the researcher male or female? | P 3-5 |
| 5. Experience & training | What experience or training did the researcher have? | HM, AA, p5 |
| *Relationship with participants* | | |
| 6. Relationship established | Was a relationship established prior to study commencement? | No relationship beyond recruitment correspondence: p 3-4 |
| 7. Participant knowledge of the interviewer | What did the participants know about the researcher? e.g. personal goals, reasons for doing the research | Participants had no information about researcher other than aims of research in participant information: p 3-4 |
| 8. Interviewer characteristics | What characteristics were reported about the inter viewer/facilitator? e.g. Bias, assumptions, reasons and interests in the research topic | Female interviewing other women, committed to biopsychosocial model of pain and to women's health: p5 |
| ***Domain 2: study design*** | | |
| *Theoretical framework* | | |
| 9. Methodological orientation and Theory | What methodological orientation was stated to underpin the study? e.g. grounded theory, discourse analysis, ethnography, phenomenology, content analysis | Design & setting: p 3 |
| *Participant selection* | | |
| 10. Sampling | How were participants selected? e.g. purposive, convenience, consecutive, snowball | Volunteers from advertisement on website of Endometriosis charity: p3 |
| 11. Method of approach | How were participants approached? e.g. face-to-face, telephone, mail, email | Email correspondence with potential participants: pp 3-4 |
| 12. Sample size | How many participants were in the study? | 16: p6 |
| 13. Non-participation | How many people refused to participate or dropped out? Reasons? | No refusals or drop-outs: p6 |
| *Setting* | | |
| 14. Setting of data collection | Where was the data collected? e.g. home, clinic, workplace | Online; participants at home: pp 3-4 |
| 15. Presence of non-participants | Was anyone else present besides the participants and researchers? | No: p3-4 |
| 16. Description of sample | What are the important characteristics of the sample? e.g. demographic data, date | 16 women: mixed ages, ethnicities, years since diagnosis. See Table 1. |
| *Data collection* | | |
| 17. Interview guide | Were questions, prompts, guides provided by the authors? Was it pilot tested? | Guide created and trained by originators of GEM; no further prompts / questions were added. Pilot by role play. Pilot on first participant generated no changes, so included in sample. Pp 3-4 |
| 18. Repeat interviews | Were repeat inter views carried out? If yes, how many? | No repeat interviews. |
| 19. Audio/visual recording | Did the research use audio or visual recording to collect the data? | Video recording of interviews kept for correction of transcription and then deleted upon completion: p4. |
| 20. Field notes | Were ﬁeld notes made during and/or after the interview or focus group? | Brief notes taken before and after interviews: p4. |
| 21. Duration | What was the duration of the inter views or focus group? | Scheduled for one hour, and most approximated one hour. |
| 22. Data saturation | Was data saturation discussed? | Sample size advised by originator of GEM; salience of themes and subthemes shown in Table 2. |
| 23. Transcripts returned | Were transcripts returned to participants for comment and/or correction? | No, but analysis checked with experts by experience: p6. |
| **Domain 3: analysis and ﬁndings** | | |
| *Data analysis* | | |
| 24. Number of data coders | How many data coders coded the data? | Two: AA and AW: p5. |
| 25. Description of the coding tree | Did authors provide a description of the coding tree? | Coding frame used after duplicate coding of two interviews: p5. |
| 26. Derivation of themes | Were themes identiﬁed in advance or derived from the data? | Derived from data: p5. |
| 27. Software | What software, if applicable, was used to manage the data? | NVivo: p5. |
| 28. Participant checking | Did participants provide feedback on the ﬁndings? | Participants did not provide feedback, but a member of staff and a volunteer from the same charity provided feedback, along with other experts by experience and clinicians: p6, Discussion, Acknowledgements. |
| *Reporting* | | |
| 29. Quotations presented | Were participant quotations presented to illustrate the themes/ﬁndings? Was each quotation identiﬁed? e.g. participant number | Yes: pp 7-14. |
| 30. Data and ﬁndings consistent | Was there consistency between the data presented and the ﬁndings? | Yes: Results pp 7-14, Discussion pp 14-15. |
| 31. Clarity of major themes | Were major themes clearly presented in the ﬁndings? | Yes, in Results pp 7-14, and in thematic map: Fig 1. |
| 32. Clarity of minor themes | Is there a description of diverse cases or discussion of minor themes? | Yes: in Results pp 7-14 and Table 2 shows salience of themes and subthemes. |
